# Pilot PET Study to Assess the Functional Interplay Between ABCB1 and ABCG2 at the Human Blood–Brain Barrier

**DOI:** 10.1002/cpt.362

**Published:** 2016-05-09

**Authors:** M Bauer, K Römermann, R Karch, B Wulkersdorfer, J Stanek, C Philippe, A Maier‐Salamon, H Haslacher, C Jungbauer, W Wadsak, W Jäger, W Löscher, M Hacker, M Zeitlinger, O Langer

**Affiliations:** ^1^ Department of Clinical PharmacologyMedical University of ViennaViennaAustria; ^2^ Department of Pharmacology, Toxicology & PharmacyUniversity of Veterinary MedicineHannoverGermany; ^3^ Center for Medical Statistics, Informatics and Intelligent SystemsMedical University of ViennaViennaAustria; ^4^ Health and Environment DepartmentAIT Austrian Institute of Technology GmbHSeibersdorfAustria; ^5^ Department of Biomedical Imaging und Image‐guided Therapy, Division of Nuclear MedicineMedical University of ViennaViennaAustria; ^6^ Department of Clinical Pharmacy and DiagnosticsUniversity of ViennaViennaAustria; ^7^ Department of Laboratory MedicineMedical University of ViennaViennaAustria; ^8^ Austrian Red Cross Blood Transfusion ServicesViennaAustria; ^9^ Medical Imaging ClusterMedical University of ViennaViennaAustria

## Abstract

ABCB1 and ABCG2 work together at the blood–brain barrier (BBB) to limit brain distribution of dual ABCB1/ABCG2 substrates. In this pilot study we used positron emission tomography (PET) to assess brain distribution of two model ABCB1/ABCG2 substrates ([^11^C]elacridar and [^11^C]tariquidar) in healthy subjects without (c.421CC) or with (c.421CA) the *ABCG2* single‐nucleotide polymorphism (SNP) c.421C>A. Subjects underwent PET scans under conditions when ABCB1 and ABCG2 were functional and during ABCB1 inhibition with high‐dose tariquidar. In contrast to the ABCB1‐selective substrate (*R*)‐[^11^C]verapamil, [^11^C]elacridar and [^11^C]tariquidar showed only moderate increases in brain distribution during ABCB1 inhibition. This provides evidence for a functional interplay between ABCB1 and ABCG2 at the human BBB and suggests that both ABCB1 and ABCG2 need to be inhibited to achieve substantial increases in brain distribution of dual ABCB1/ABCG2 substrates. During ABCB1 inhibition c.421CA subjects had significantly higher increases in [^11^C]tariquidar brain distribution than c.421CC subjects, pointing to impaired cerebral ABCG2 function.

**Study Highlights**

**WHAT IS THE CURRENT KNOWLEDGE ON THE TOPIC?**

☑ Preclinical data in rodents suggest that Abcb1a and Abcg2 work together at the BBB in limiting brain distribution of ABCB1/ABCG2 substrate drugs.

**WHAT QUESTION DID THIS STUDY ADDRESS?**

☑ Our pilot study examined for the first time the functional interplay between ABCB1 and ABCG2 at the human BBB by assessing brain distribution of the model ABCB1/ABCG2 substrates [^11^C]elacridar and [^11^C]tariquidar with PET under conditions when ABCB1 and ABCG2 were functional and during ABCB1 inhibition, in subjects who were either noncarriers or heterozygous carriers of the *ABCG2* SNP c.421C>A.

**WHAT THIS STUDY ADDS TO OUR KNOWLEDGE**

☑ We obtained first evidence for a functional interplay between ABCB1 and ABCG2 at the human BBB resulting in only moderate increases in brain distribution of ABCB1/ABCG2 substrates when ABCB1 alone is inhibited. However, in c.421CA subjects the effects of ABCB1 inhibition were more pronounced than in c.421CC subjects.

**HOW THIS MIGHT CHANGE CLINICAL PHARMACOLOGY AND THERAPEUTICS**

☑ Our data suggest an overall low risk for transporter‐mediated drug–drug interactions for ABCB1/ABCG2 substrate drugs, which may be higher in carriers of the c.421C>A SNP.

The adenosine triphosphate‐binding cassette (ABC) transporters P‐glycoprotein (humans: ABCB1, rodents: Abcb1a) and breast cancer resistance protein (humans: ABCG2, rodents: Abcg2) are two major efflux transporters expressed at the luminal membrane of brain capillary endothelial cells forming the blood–brain barrier (BBB).^1^ They can restrict distribution of their substrates from blood to the brain by efflux transport directed from the endothelial cell back into blood. Several clinically used drugs are substrates of both ABCB1 and ABCG2 including most tyrosine kinase inhibitors, some other anticancer drugs,^2^, ^3^ as well as drugs for other central nervous system (CNS) indications.^4^, ^5^, ^6^ ABCB1/ABCG2 efflux transport generally results in very low brain exposure of drugs.

Studies in transporter knockout mice have shown that Abcb1a and Abcg2 work together in limiting brain distribution of shared (dual) ABCB1/ABCG2 substrates.^7^ When only Abcb1a alone or Abcg2 alone is knocked out, the other transporter restricts brain access of dual substrates, so that dual substrates can only gain brain access when both Abcb1a and Abcg2 are knocked out. This functional interplay between Abcb1a and Abcg2 at the BBB has been clearly demonstrated in mice for several dual ABCB1/ABCG2 substrates, such as mitoxantrone, erlotinib, lapatinib, and topotecan.^7^, ^8^, ^9^ It is, however, to date not known to what extent the functional interplay between Abcb1a and Abcg2 observed at the rodent BBB translates to the human BBB. This is of particular interest given recent evidence for pronounced species differences in relative expression levels of ABCB1 and ABCG2 at the rodent and human BBB with ABCG2/ABCB1 expression ratios of ∼1.3 in humans and 0.3 in mice.^10^, ^11^, ^12^ This may potentially lead to species differences in brain distribution of dual ABCB1/ABCG2 substrates as well as to different susceptibilities to transporter‐mediated drug–drug interactions (DDIs) at the BBB.^1^

Positron emission tomography (PET) is a noninvasive nuclear imaging technique that enables assessment of brain distribution of drugs labeled with positron‐emitting radionuclides, such as carbon‐11 (^11^C) or fluorine‐18 (^18^F).^13^ PET has been used to study ABCB1 function at the human BBB including the assessment of ABCB1‐mediated DDIs.^14^, ^15^, ^16^, ^17^, ^18^ However, so far dual ABCB1/ABCG2 substrate radiotracers have not been examined in humans. We and others have recently shown that the radiolabeled ABCB1 inhibitors [^11^C]elacridar and [^11^C]tariquidar are transported at microdoses by Abcb1a and Abcg2 at the rodent BBB, while lacking brain distribution of radiolabeled metabolites.^19^, ^20^, ^21^, ^22^, ^23^, ^24^ In addition, there is evidence that elacridar and tariquidar are also substrates of human ABCB1 and ABCG2.^22^, ^25^, ^26^

The aim of the present pilot study was to use [^11^C]elacridar and [^11^C]tariquidar as two model substrates to assess for the first time the functional interplay between ABCB1 and ABCG2 at the human BBB. We studied brain distribution of both probe substrates with PET under conditions when both ABCB1 and ABCG2 were fully functional, as well as during infusion of unlabeled tariquidar at a dose that inhibits ABCB1 without inhibiting ABCG2,^21^ so that the specific contribution of ABCG2 could be revealed. Moreover, the effect of a well‐known nonsynonymous single‐nucleotide polymorphism (SNP) in the *ABCG2* gene (c.421C>A), which has been associated with reduced ABCG2 function,^27^, ^28^ was studied on [^11^C]tariquidar brain distribution.

## RESULTS

### *In vitro* transport assays

**Figure 1:**
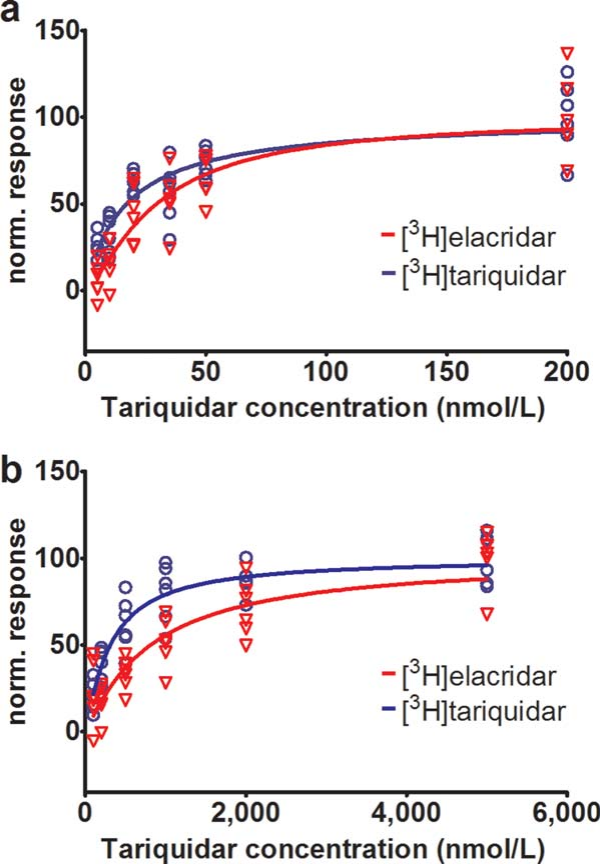
Normalized response to ABCB1 or ABCG2 inhibition for [^3^H]elacridar (red) and [^3^H]tariquidar (blue) transport in LLC‐ABCB1 cells (**a**) or MDCK‐ABCG2 cells (**b**) is plotted against tariquidar concentration (nmol/L) in the assay medium. Shown data are from one experiment performed with six technical replicates for each cell line. A sigmoidal Hill function was fitted to the data and gave for uptake in ABCB1 cells an estimated half–maximum inhibitory concentration (IC_50_) of 29.5 nmol/L (95% confidence interval (CI): 23.7–36.8) and a Hill slope of 1.4 (95% CI: 0.9–1.8) for [^3^H]elacridar as substrate, and an IC_50_ of 17.1 nmol/L (95% CI: 13.3–21.9) and a Hill slope of 1.0 (95% CI: 0.7–1.3) for [^3^H]tariquidar as substrate (**a**). For uptake in ABCG2 cells an IC_50_ of 812.9 nmol/L (95% CI: 620.5–1,065.0) and a Hill slope of 1.1 (95% CI: 0.8–1.4) were estimated for [^3^H]elacridar as substrate, and for [^3^H]tariquidar as substrate an IC_50_ of 310.4 nmol/L (95% CI: 251.0–383.9) and a Hill slope of 1.1 (95% CI: 0.9–1.4) (**b**). For definition of normalized response refer to the Methods section.

We performed uptake experiments in cell lines overexpressing either human ABCB1 (**Figure** 1 **a**) or ABCG2 (**Figure** 1 **b**) to determine the inhibitory effect of tariquidar on ABCB1‐ and ABCG2‐mediated transport of [^3^H]elacridar and [^3^H]tariquidar. In both cell lines cellular uptake of [^3^H]elacridar and [^3^H]tariquidar increased with increasing concentrations of unlabeled tariquidar in the medium (**Figure** 1). Half‐maximum inhibitory concentrations (IC_50_) of tariquidar for inhibition of ABCB1 transport were 28‐fold and 18‐fold lower than for inhibition of ABCG2 transport for [^3^H]elacridar and [^3^H]tariquidar, respectively. This large difference in IC_50_ values supports that the tariquidar dose administered during our PET study inhibits ABCB1 and not ABCG2 at the BBB.

### PET study

Study participants who were either noncarriers (c.421CC) or heterozygous carriers (c.421CA) of the *ABCG2* c.421C>A SNP underwent two consecutive PET scans with either [^11^C]elacridar or [^11^C]tariquidar, a first baseline scan, and a second scan during continuous i.v. infusion of unlabeled tariquidar. Five c.421CC subjects and one c.421CA subject received [^11^C]elacridar and five c.421CC subjects, six c.421CA subjects, and one c.421AA subject received [^11^C]tariquidar. In the c.421AA subject only the first PET scan with [^11^C]tariquidar could be completed due to expiry of the study medication tariquidar. One c.421CA subject discontinued the [^11^C]tariquidar baseline PET scan due to claustrophobia. So we report in total [^11^C]elacridar PET data in five c.421CC subjects and one c.421CA subject and [^11^C]tariquidar PET data in five c.421CC subjects, five c.421CA subjects, and one c.421AA subject. Adverse events occurring during study participation for which a relationship with tariquidar administration could not be excluded were dysgeusia (10 subjects), collapse (four subjects), phlebitis (three subjects), headache (three subjects), dizziness (two subjects), and abdominal cramps and vomiting (one subject).

### PET in c.421CC subjects

**Figure 2:**
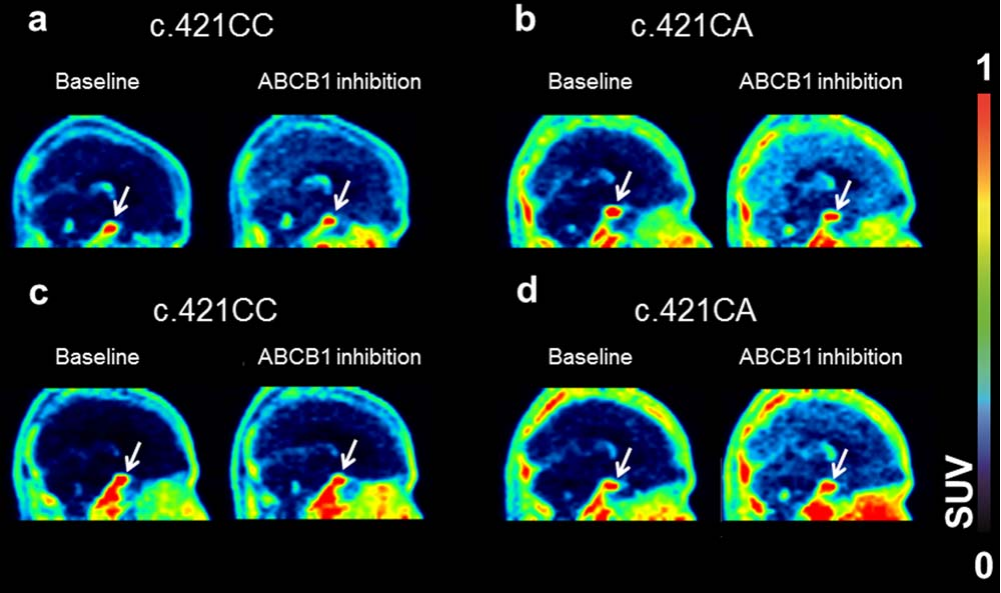
Sagittal PET summation images (0–60 min) for [^11^C]elacridar (**a,b**) and [^11^C]tariquidar (**c,d**) in c.421CC and c.421CA subjects for baseline scans and scans during ABCB1 inhibition. In **b** and **d**, an identical c.421CA subject is shown. Radioactivity concentration was normalized to injected radioactivity amount per body weight and expressed as standardized uptake value (SUV) and radiation scale is set from 0 to 1.0. Pituitary gland is labeled with white arrow.

**Figure 3:**
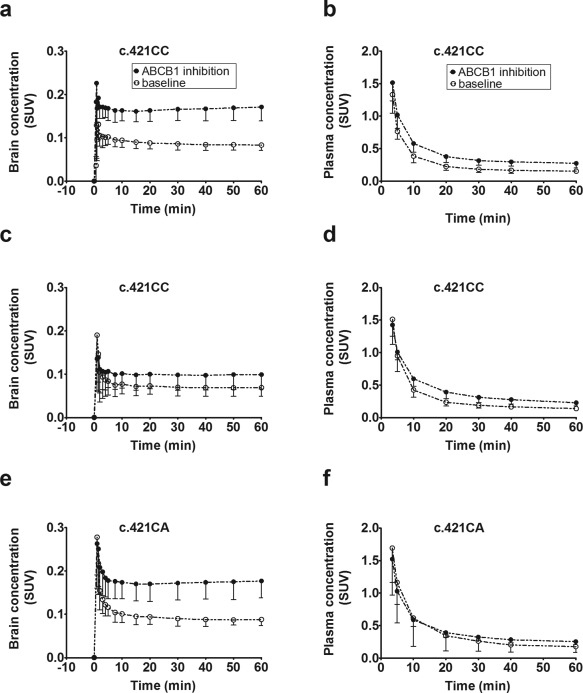
Mean time–activity curves (standardized uptake value, SUV ± SD) of [^11^C]elacridar (**a,b**) and [^11^C]tariquidar (**c–f**) in whole brain gray matter (**a,c,e**) and arterial plasma (**b,d,f**) for baseline scans (i.e., without ABCB1 inhibition) and scans during ABCB1 inhibition in c.421CC and c.421CA subjects (*n* = 5 per group). Brain time‐activity curves were corrected for radioactivity in the vasculature by subtraction of total radioactivity counts in arterial blood, scaled to 5% (vascular contribution to total brain volume). The same figure with log‐linear axis is provided as **Supplementary Figure 2**.

In **Figure** 2 representative PET images of [^11^C]elacridar and [^11^C]tariquidar in c.421CC subjects are shown. Both radiotracers showed very low brain uptake. In plasma, only a low amount of circulating radiolabeled metabolites was detected during the PET scans, both for [^11^C]elacridar and [^11^C]tariquidar. In the baseline scans the percentage of unchanged radiotracer in plasma at 60 minutes after injection was 94.6 ± 2.3% for [^11^C]elacridar and 89.7 ± 3.6% for [^11^C]tariquidar. In the second PET scan this value was not significantly different from the first scan for [^11^C]elacridar (94.7 ± 1.1%) and [^11^C]tariquidar (91.1 ± 2.7%). Plasma protein binding of [^11^C]elacridar and [^11^C]tariquidar appeared to be very high (>99%) and similar in the baseline and ABCB1 inhibition scans. However, the plasma free fractions of [^11^C]elacridar and [^11^C]tariquidar could not be reliably determined due to a high degree of nonspecific binding of both radiotracers to the filter membranes of the ultrafiltration devices. Plasma concentrations of [^11^C]elacridar and [^11^C]tariquidar were significantly (*P* = 0.043) increased during tariquidar infusion as compared with baseline PET scans (**Figure** 3 **b,d**). AUCs for plasma time–activity curves (TACs) in scans 1 and 2 are provided in **Supplementary Table 1**. For both radiotracers, brain concentrations of radioactivity were increased during ABCB1 inhibition (**Figure** 3 **a,c**). Data were analyzed using noncompartmental and compartmental modeling approaches. For compartmental modeling, a 2‐tissue‐4‐rate constant (2T4K) compartment model was used, which we have shown in our previous work to provide better fits of the data than a 1‐tissue‐2‐rate constant (1T2K) compartment model.^26^ For [^11^C]elacridar, the ratio of brain to plasma AUC (AUCR), total distribution volume (*V*_T_) calculated with Logan graphical analysis (*V*_T_ (Logan)), influx rate constant of radioactivity from plasma into brain (*K*_1_) and *V*_T_ calculated with 2T4K model (*V*_T_ (2T4K)) were significantly increased in scan 2 as compared with scan 1 (**Table** 1). For [^11^C]tariquidar, none of the outcome parameters was significantly changed in scan 2 as compared with scan 1 (**Table** 2). Tariquidar plasma concentrations during the PET scan (average of three determinations at beginning, middle, and end of PET scan) were 2.6 ± 0.5 μmol/L for the [^11^C]elacridar group and 2.6 ± 1.1 μmol/L for the [^11^C]tariquidar group (**Supplementary Table 2**). There was for both radiotracers no significant correlation between changes in outcome parameters in scan 2 and tariquidar plasma concentration at time of PET.

**Table 1:** [^11^C]Elacridar outcome parameters (whole brain) in c.421CC subjects for baseline scan and scan during ABCB1 inhibition with tariquidar

Group	AUCR	*V*_T_ (Logan)	*K*_1_ (mL/(g x min))	*k*_2_ (1/min)	*k*_3_ (1/min)	*k*_4_ (1/min)	*V*_T_ (2T4K)
c.421CC baseline	0.179 ± 0.027	0.478 ± 0.027 (6)	0.006 ± 0.002 (9)	0.135 ± 0.050 (33)	0.148 ± 0.040 (22)	0.015 ± 0.004 (17)	0.492 ± 0.051 (9)
c.421CC ABCB1 inhibition	0.245 ± 0.027*	0.658 ± 0.098 (9)*	0.013 ± 0.005 (8)*	0.302 ± 0.250 (31)	0.198 ± 0.050 (21)	0.016 ± 0.003 (13)	0.676 ± 0.111 (6)*

Outcome parameters are given as mean ± standard deviation averaged over all subjects (*n* = 5) per group. The value in parentheses represents the precision of parameter estimates (expressed as their coefficient of variation in percent), averaged over all subjects per group.

AUCR, ratio of brain to plasma area under the time‐activity curve (0–60 min); *K*_1_, *k*_2_, *k*_3_, *k*_4_, rate constants for transfer of activity between the plasma, the first and the second tissue compartments calculated with 2‐tissue‐4‐rate constant (2T4K) compartment model; *V*_T_, total distribution volume; *V*_T_ (Logan), *V*_T_ calculated with Logan graphical analysis; *V*_T_ (2T4K), *V*_T_ calculated with 2T4K model.

*P < 0.05 for comparison with baseline scan using Wilcoxon matched‐pairs signed rank test.

**Table 2:** [^11^C]Tariquidar outcome parameters (whole brain) in c.421CC and c.421CA subjects for baseline scan and scan during ABCB1 inhibition with tariquidar

Group	AUCR	*V*_T_ (Logan)	*K*_1_ (mL/(g x min))	*k*_2_ (1/min)	*k*_3_ (1/min)	*k*_4_ (1/min)	*V*_T_ (2T4K)
c.421CC baseline	0.163 ± 0.054	0.430 ± 0.102 (8)	0.009 ± 0.004 (26)	0.340 ± 0.209 (57)	0.152 ± 0.037 (34)	0.012 ± 0.006 (23)	0.450 ± 0.089 (16)
c.421CC ABCB1 inhibition	0.173 ± 0.051	0.408 ± 0.090 (7)	0.008 ± 0.002 (16)	0.176 ± 0.103 (46)	0.119 ± 0.050 (32)	0.014 ± 0.005 (25)	0.566 ± 0.285 (15)
c.421CA baseline	0.186 ± 0.033	0.416 ± 0.111 (13)	0.008 ± 0.002 (32)	0.193 ± 0.074 (70)	0.116 ± 0.055 (38)	0.010 ± 0.001 (41)	0.533 ± 0.146 (24)
c.421CA ABCB1 inhibition	0.273 ± 0.048*	0.738 ± 0.196 (8)*	0.013 ± 0.004 (13)*	0.195 ± 0.097 (34)	0.140 ± 0.040 (22)	0.015 ± 0.006 (18)	0.770 ± 0.207 (10)

Outcome parameters are given as mean ± standard deviation averaged over all subjects (*n* = 5) per group. The value in parentheses represents the precision of parameter estimates (expressed as their coefficient of variation in percent), averaged over all subjects per group.

AUCR, ratio of brain to plasma area under the time‐activity curve (0–60 min); *K*_1_, *k*_2_, *k*_3_, *k*_4_, rate constants for transfer of activity between the plasma, the first and the second tissue compartments calculated with 2‐tissue‐4‐rate constant (2T4K) compartment model; *V*_T_, total distribution volume; *V*_T_ (Logan), *V*_T_ calculated with Logan graphical analysis; *V*_T_ (2T4K), *V*_T_ calculated with 2T4K model.

*P < 0.05 for comparison with baseline scan using Wilcoxon matched‐pairs signed rank test.

### PET in c.421CA subjects

**Figure 4:**
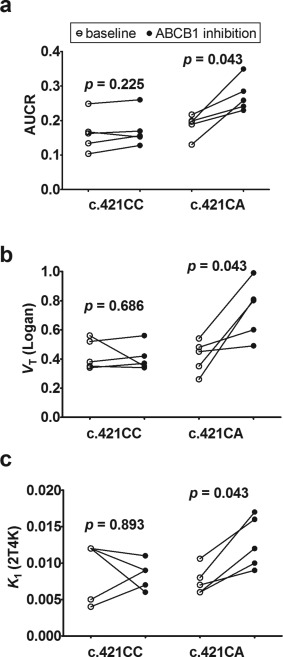
Ratio of brain to plasma area under the time–activity curve (AUCR) (**a**), *V*_T_ (calculated with Logan graphical analysis) (**b**), and *K*_1_ calculated from 2T4K model (**c**) of [^11^C]tariquidar in c.421CC and c.421CA subjects in baseline and ABCB1 inhibition scans. *P* values for comparison of baseline with ABCB1 inhibition scans using Wilcoxon matched‐pairs signed rank test are shown in the graph.

For [^11^C]tariquidar, PET imaging was additionally performed in a group of subjects with the c.421CA genotype. In addition, one female subject with the c.421AA genotype underwent a [^11^C]tariquidar baseline PET scan. In the baseline scan, *V*_T_ (Logan) values of c.421CA subjects (0.42 ± 0.11) were not significantly different from c.421CC subjects (0.43 ± 0.10) (**Table** 2). *V*_T_ (Logan) of the c.421AA subject was 0.40 (data not shown in **Table** 2), which was comparable with *V*_T_ (Logan) values in c.421CC and c.421CA subjects. In scan 2, *V*_T_ (Logan) values of c.421CA subjects were significantly higher than for c.421CC subjects (0.74 ± 0.20 vs. 0.41 ± 0.09, *P* = 0.016) (**Table** 2). In c.421CA subjects, AUCR, *V*_T_ (Logan), and *K*_1_ were significantly increased in scan 2 as compared with scan 1, whereas *V*_T_ (2T4K) showed a trend for increases without reaching statistical significance (*P* = 0.144) (**Figure** 4, **Table** 2). Scan 2/scan 1 ratios of AUCR (*P* = 0.032) and *V*_T_ (Logan) (*P* = 0.016) were significantly different between c.421CC and c.421CA subjects. In c.421CC subjects, *V*_T_ (Logan) in scan 2 was essentially unchanged as compared with scan 1 (scan 2/scan 1 ratio: 1.0 ± 0.2). In contrast, in three out of five c.421CAsubjects pronounced increases (1.8‐fold to 3.1‐fold) in *V*_T_ (Logan) were observed in scan 2 relative to scan 1, whereas two c.421CA subjects had smaller increases in *V*_T_ (Logan) (1.1‐fold to 1.3‐fold). Mean scan 2/scan 1 ratio of *V*_T_ (Logan) in c.421CA subjects was 1.9 ± 0.8. Tariquidar plasma concentrations at the time of PET were not significantly different between subjects with the c.421CC (2.6 ± 1.1 μmol/L) and the c.421CA genotype (3.2 ± 0.4 μmol/L) (**Supplementary Table 2**). Moreover, there was no significant correlation between scan 2/scan 1 AUCR and *V*_T_ (Logan) ratios and tariquidar plasma concentrations, neither in c.421CA subjects nor in c.421CC subjects nor in both groups combined. In addition, we analyzed the PET data from 0–10 minutes with a 1T2K model, which showed in c.421CA subjects significant increases in *K*_1_ (*P* = 0.043) and significant decreases in the efflux rate constant of radioactivity from brain to plasma *k*_2_ (*P* = 0.043) during tariquidar infusion (**Supplementary Figure 1**). Moreover, *K*_1_/*k*_2_ increases in the ABCB1 inhibition scan relative to the baseline scan were significantly greater in c.421CA (2.2 ± 0.6‐fold) than in c.421CC subjects (1.4 ± 0.2‐fold, *P* = 0.036). The c.421CA subject with the highest increase in [^11^C]tariquidar *V*_T_ (Logan) in scan 2 (3.1‐fold increase) was rescanned with [^11^C]elacridar (**Figure** 2). This subject also had for [^11^C]elacridar a markedly higher increase in *V*_T_ (Logan) in scan 2 (2.3‐fold increase, data not shown) as compared with the mean increase in [^11^C]elacridar *V*_T_ (Logan) in c.421CC subjects (1.4 ± 0.2‐fold increase).

### Comparison of brain distribution with pituitary gland

**Figure 5:**
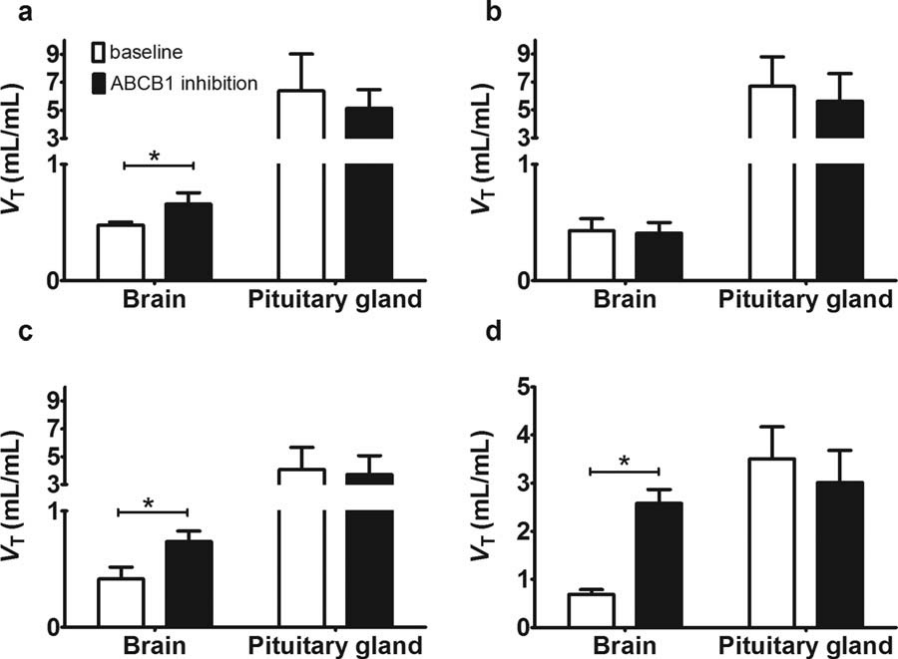
*V*_T_ values (calculated with Logan graphical analysis) in whole brain gray matter and pituitary gland for baseline scans (i.e., without ABCB1 inhibition) and scans with ABCB1 inhibition for [^11^C]elacridar in c.421CC subjects (**a**), for [^11^C]tariquidar in c.421CC subjects (**b**), for [^11^C]tariquidar in c.421CA subjects (**c**), and for the ABCB1‐selective substrate (*R*)‐[^11^C]verapamil (**d**, data taken from Ref. 18) (*n* = 5 for each group). For all three radiotracers an identical tariquidar administration protocol was used, which afforded comparable tariquidar plasma concentrations (see **Supplementary Table 2**). (*P < 0.05, Wilcoxon matched‐pairs signed rank test).

The pituitary gland, a brain region with fenestrated capillaries, which is not protected by the BBB, was also outlined on the PET images (**Figure** 2). Both for [^11^C]elacridar and [^11^C]tariquidar, *V*_T_ (Logan) values in the pituitary gland were not significantly different during tariquidar infusion as compared with baseline scans. We then compared *V*_T_ (Logan) values of [^11^C]elacridar and [^11^C]tariquidar in whole brain gray matter with those of the pituitary gland (**Figure** 5). For [^11^C]elacridar, whole brain *V*_T_ (Logan) values were 13.2 ± 4.6‐fold and 7.9 ± 2.1‐fold lower than *V*_T_ (Logan) values in the pituitary gland in scan 1 and scan 2, respectively (**Figure** 5 **a**). For [^11^C]tariquidar, whole brain *V*_T_ (Logan) values in c.421CC subjects were 15.9 ± 4.9‐fold and 13.9 ± 4.7‐fold lower than *V*_T_ (Logan) values in the pituitary gland in scan 1 and scan 2, respectively (**Figure** 5 **b**). In c.421CA subjects, [^11^C]tariquidar whole brain *V*_T_ (Logan) values were 9.5 ± 1.6‐fold and 5.4 ± 2.9‐fold lower than *V*_T_ (Logan) values in the pituitary gland in scan 1 and scan 2, respectively (**Figure** 5 **c**). This contrasted with previously published data for the ABCB1‐selective radiotracer (*R*)‐[^11^C]verapamil,^18^ for which whole brain *V*_T_ (Logan) was 5.2 ± 1.4‐fold lower than in the pituitary gland in scan 1, but only 1.2 ± 0.2‐fold lower than in the pituitary gland in scan 2 (**Figure** 5 **d**). Based on the data shown in **Figure** 5 we also calculated fraction transported (f_t_) for all three radiotracers as described before (**Supplementary Table 3**).^29^

## DISCUSSION

This is to our knowledge the first study in which the functional interplay between ABCB1 and ABCG2 was assessed *in vivo* at the human BBB. We used PET together with [^11^C]elacridar and [^11^C]tariquidar as two metabolically stable model compounds, for which it has been previously shown that they are at low concentrations as employed in PET experiment substrates of murine and human ABCB1 and ABCG2.^22^, ^25^, ^26^ Here we confirmed by performing *in vitro* uptake experiments in cell lines overexpressing human ABCB1 or ABCG2 that both compounds are substrates of human ABCB1 and ABCG2. Moreover, we demonstrate that increasing doses of tariquidar inhibit ABCB1‐mediated transport of both probe substrates more potently than ABCG2‐mediated transport (**Figure** 1). *In vitro* IC_50_ values of tariquidar for inhibition of ABCB1 transport of [^3^H]elacridar (29.5 nmol/L) and [^3^H]tariquidar (17.1 nmol/L) were comparable with the previously determined IC_50_ value for inhibition of ABCB1 transport of [^3^H]verapamil (17.2 nmol/L), using the same cell line,^30^ which suggested that tariquidar inhibited ABCB1 transport of all three substrates with comparable potency.

We then characterized both radiotracers *in vivo* in healthy human subjects by employing a recently developed PET protocol involving two consecutive scans, a first baseline scan and a second scan during ABCB1 inhibition achieved by coinfusion of tariquidar.^18^ We have shown in a previous study with the ABCB1‐selective radiotracer (*R*)‐[^11^C]verapamil that this continuous‐infusion tariquidar administration protocol leads to almost complete inhibition of ABCB1 at the human BBB,^18^ without affecting cerebral blood flow.^15^ Plasma concentrations of tariquidar at the time of the second PET scan were above the previously estimated *in vivo* IC_50_ value for inhibition of ABCB1 transport of (*R*)‐[^11^C]verapamil at the human BBB (2.3 ± 1.0 μmol/L),^18^ which suggested that a substantial degree of ABCB1 inhibition was achieved in our study. Tariquidar is the only currently available ABCB1 inhibitor that can be used in humans to achieve such a high degree of ABCB1 inhibition at the BBB.^16^, ^18^ However, a limitation of tariquidar restricting its wider use is that it is a nonmarketed drug with limited availability, which is only available as an i.v. infusion solution with a short shelf life. Based on our *in vitro* data that revealed 28‐fold and 18‐fold lower IC_50_ values of tariquidar for inhibition of ABCB1 transport than for inhibition of ABCG2 transport of [^3^H]elacridar and [^3^H]tariquidar, respectively (**Figure** 1), it can be expected that the employed tariquidar dose did not inhibit ABCG2 transport of [^11^C]elacridar and [^11^C]tariquidar at the human BBB.

Brain distribution of both probe substrates was very low (**Figure** 2), which was consistent with efflux transport by ABCB1 and ABCG2. Both radiotracers showed only a low amount (<10%) of radiolabeled metabolites in plasma, which suggested good metabolic stability in humans, like in rodents.^19^, ^20^ During tariquidar infusion, both radiotracers showed a significant increase in plasma exposure (**Figure** 3, **Supplementary Table 1**), which may have been caused by inhibition of ABCB1 and/or other transporters in clearance organs like the liver. Both radiotracers showed increases in brain exposure during ABCB1 inhibition, but this was partly due to increased plasma exposure (**Figure** 3). PET data were modeled by using previously described noncompartmental and compartmental modeling approaches,^26^, ^31^ which revealed only for [^11^C]elacridar moderate (1.4‐fold) increases in brain distribution during ABCB1 inhibition. AUCR, *V*_T_ (Logan), *V*_T_ (2T4K), and *K*_1_ were significantly increased for [^11^C]elacridar in scan 2 as compared with scan 1 (**Table** 1). These were the same modeling outcome parameters that were previously shown to be affected by ABCB1 inhibition for the ABCB1‐selective radiotracers racemic [^11^C]verapamil, (*R*)‐[^11^C]verapamil, and [^11^C]*N*‐desmethyl‐loperamide.^15^, ^17^, ^32^ Importantly, both [^11^C]elacridar and [^11^C]tariquidar showed reversible kinetics, as reflected by an exchange rate constant from the second to the first brain tissue compartment *k*_4_ > 0 (**Table** 1 and **2**). For [^11^C]tariquidar no significant increases in modeling outcome parameters reflecting BBB passage were observed during ABCB1 inhibition (**Table** 2). We hypothesize that this is due to the fact that brain distribution of [^11^C]tariquidar is less dependent on ABCB1 function than that of [^11^C]elacridar. This assumption is supported by data in transporter knockout mice, which showed higher increases in brain distribution for [^11^C]elacridar (3.1‐fold) than for [^11^C]tariquidar (1.9‐fold) in *Abcb1a/b^(‐/‐)^* mice as compared with wildtype mice.^21^, ^22^ The markedly higher increases in brain distribution of [^11^C]elacridar and [^11^C]tariquidar in the absence of Abcb1a function observed in mice 21, 22 are in line with the relatively higher abundance of Abcb1a in the mouse as compared with the human BBB.^10^, ^12^ In comparison with a previous preclinical study,^31^ baseline whole brain *V*_T_ (Logan) values of [^11^C]elacridar and [^11^C]tariquidar were ∼2‐fold lower in humans than in rats, which could be related to differences in ABCB1/ABCG2 expression levels in brain capillaries of rats and humans.^12^, ^33^

A direct assessment of the influence of ABCG2 function on brain distribution of [^11^C]tariquidar by employing a chemical inhibitor of ABCG2 (e.g., Ko143), as we have done in a preclinical study,^21^ was not possible in humans due to the lack of a clinically usable ABCG2 inhibitor. In order to overcome this limitation we studied a group of volunteers who were heterozygous carriers of the *ABCG2* c.421C>A SNP. Previous studies have demonstrated that this genetic polymorphism leads to diminished expression of ABCG2 in various tissues, such as the liver, placenta, and intestine,^34^, ^35^ and can also lead to changes in plasma pharmacokinetics of ABCG2 substrate drugs, such as sulfasalazine and rosuvastatin.^27^, ^28^ It has been estimated that *in vivo* intestinal ABCG2 transport activity in c.421AA subjects is ∼23% of that in c.421CC subjects.^36^ Among Caucasians, homozygous carriers of the c.421C>A SNP are very rare (∼1%), but heterozygous carriers of the variant allele are more common (∼17%).^28^ Brain distribution of [^11^C]tariquidar did not differ between c.421CA and c.421CC subjects in baseline scans, in which both ABCB1 and ABCG2 were functional (**Table** 2). We additionally examined one person with the c.421AA genotype, which is expected to lead to a greater reduction in ABCG2 expression as compared with the c.421CA genotype.^27^, ^28^ This subject showed no difference in [^11^C]tariquidar brain distribution in the baseline scan when compared to c.421CA and c.421CC subjects. This suggests that under conditions when both ABCB1 and ABCG2 are functional the effect of the *ABCG2* SNP is masked by ABCB1 function. However, under conditions of ABCB1 inhibition, increases in brain distribution of [^11^C]tariquidar were significantly greater in c.421CA than in c.421CC subjects, suggesting impaired ABCG2 function at the BBB of c.421CA subjects (**Figure** 4). This is to our knowledge the first evidence for an effect of the *ABCG2* c.421C>A SNP on ABCG2 transport activity at the human BBB. Three out of five c.421CA subjects showed markedly higher increases in [^11^C]tariquidar brain distribution during ABCB1 inhibition than the c.421CC subjects (**Figure** 4). In two out of five c.421CA subjects, increases in brain distribution were less pronounced, which, however, could not be explained by lower tariquidar plasma concentrations in these two subjects as compared with the other three subjects (see **Supplementary Table 2**, subjects 1 and 2). Apart from a significant increase in *K*_1_, the 1T2K model also revealed a significant decrease in *k*_2_ of [^11^C]tariquidar in c.421CA subjects in response to tariquidar (**Supplementary Figure 1)**. Such an effect has also been observed for other ABCB1 substrate PET tracers.^37^

The c.421CA subject with the highest increase in [^11^C]tariquidar brain distribution following ABCB1 inhibition was rescanned with [^11^C]elacridar, for which a markedly higher increase in brain distribution was also observed in scan 2 as compared with the c.421CC group (**Figure** 2). Even though rescanning with [^11^C]elacridar could be done in only one subject, our data provide evidence that the effect of the *ABCG2* SNP is not specific to [^11^C]tariquidar as a dual ABCB1/ABCG2 probe substrate.

In contrast to (*R*)‐[^11^C]verapamil as radiotracer, for which we were able to study brain distribution in humans in the almost complete absence of efflux transport at the BBB,^18^ we were not able to study brain distribution of [^11^C]elacridar and [^11^C]tariquidar in the complete absence of ABCB1 and ABCG2 function. This would require a potent dual ABCB1/ABCG2 inhibitor such as elacridar, which is currently not available for use in humans.^38^ To obtain an estimate of maximum possible brain distribution of [^11^C]elacridar and [^11^C]tariquidar in the absence of ABCB1 and ABCG2 function, we studied, in analogy to previous studies with other radiotracers,^16^, ^18^, ^39^ distribution to the pituitary gland, a brain region with fenestrated capillaries that is not protected by the BBB.^40^ We have recently shown that distribution of (*R*)‐[^11^C]verapamil to the pituitary gland is comparable to its brain distribution when ABCB1 is almost completely inhibited (**Figure** 5 **d**).^18^ Both for [^11^C]elacridar and [^11^C]tariquidar the pituitary gland was clearly visible in the PET images and showed markedly higher radioactivity uptake as compared with the rest of the brain (**Figure** 2). It should be noted, however, that the pituitary gland is a small structure, which may be vulnerable to partial volume effects, which could lead to inaccuracies in measuring its true radioactivity concentration. Under conditions of ABCB1 inhibition brain uptake of [^11^C]elacridar and [^11^C]tariquidar was still 7.9 ± 2.1‐fold and 13.9 ± 4.7‐fold lower than uptake in the pituitary gland, implying that maximum possible brain uptake was not reached (**Figure** 5). This suggests that ABCG2 effectively compensated ABCB1 function at the BBB, thereby restricting brain distribution of both probe substrates during ABCB1 inhibition. The moderate increases in brain distribution of [^11^C]elacridar and [^11^C]tariquidar during ABCB1 inhibition contrasts with the behavior of the ABCB1‐selective radiotracer (*R*)‐[^11^C]verapamil, for which brain uptake was 3.8 ± 0.6‐fold increased—reaching similar levels as in the pituitary gland—when ABCB1 was inhibited using an identical tariquidar administration protocol (**Figure** 5 **d**).^18^ Our data provide the first evidence that a functional interplay between ABCB1 and ABCG2, as described before in rodents,^7^, ^8^, ^9^ also exists at the human BBB, providing an effective protection system of the human brain. Increases in brain distribution of the two studied ABCB1/ABCG2 substrates following ABCB1 inhibition were considerably smaller in humans as compared with rodents, which is in good agreement with previously reported species differences in ABCG2/ABCB1 expression ratios at the BBB.^12^ A significant enhancement of the distribution of dual ABCB1/ABCG2 substrates to the human brain only seems to be possible when both ABCB1 and ABCG2 are simultaneously inhibited. This clearly diminishes the risk for clinically relevant transporter‐mediated DDIs at the human BBB for dual ABCB1/ABCG2 substrate drugs, because perpetrator drugs, which potently inhibit both transporters at clinical plasma concentrations, are expected to be very rare.^1^ However, our pilot data suggest that carriers of the c.421C>A SNP may be more vulnerable to ABCB1‐mediated DDIs involving dual ABCB1/ABCG2 substrate drugs than subjects without the *ABCG2* SNP, although this needs to be confirmed with additional substrates in larger cohorts.

A limitation of our study is that we were most likely not able to achieve complete ABCB1 inhibition with the employed tariquidar dose, resulting in some remaining ABCB1 transport activity during the second PET scan. However, due to safety concerns it was not possible to administer higher doses of tariquidar in our study.

In conclusion, our pilot study provides the first evidence for a functional interplay between ABCB1 and ABCG2 at the human BBB in limiting brain distribution of dual ABCB1/ABCG2 substrate drugs, which is expected to result in a very low risk for transporter‐mediated DDIs for such drugs. The PET protocol employed in the present study allowed for the first time measuring the transport activity of ABCG2 at the human BBB and might also be applicable to investigate cerebral ABCG2 function in other settings, such as in different neurological diseases.

## METHODS

Our study was conducted as a pilot study, was registered with EUDRACT (number 2012‐005796‐14), approved by the Ethics Committee of the Medical University of Vienna, and conducted in accordance with the current (2013) version of the Declaration of Helsinki. All subjects were given a detailed description of the study, and their written consent was obtained before entry into the study. Study participants were defined as healthy based on medical history, physical examination, routine blood and urine laboratory testing, and were required to be free of any medication for at least 14 days before start of the study.

### Genotyping

Four‐mL venous blood samples were drawn from all study participants. Subjects were genotyped for the *ABCG2* c.421C>A SNP by means of probe‐based polymerase chain reaction (PCR) applying the TaqMan method.^41^ We were able to identify 11 subjects with the c.421CA genotype from 52 screened subjects. In addition, one female subject with the c.421AA genotype was recruited from an existing database at the Austrian Red Cross Blood Transfusion Services. In total, 10 male subjects with the c.421CC genotype, six male subjects with the c.421CA genotype, and one female subject with the c.421AA genotype participated in the PET imaging part of the study.

### Radiotracer synthesis

[^11^C]Elacridar and [^11^C]tariquidar were synthesized following previously published procedures.^19^, ^20^ Radiotracers were formulated in sterile 0.9% (w/v) aqueous saline solution/ethanol (9/1, v/v) containing 0.7% (v/v) polysorbate‐80. Specific activities at the time of injection were 41 ± 15 GBq/μmol for [^11^C]elacridar (*n* = 12 batches) and 58 ± 37 GBq/μmol for [^11^C]tariquidar (*n* = 22 batches). Radiochemical purity of all radiotracer batches was >95%.

### Safety monitoring

During the PET study, heart rate and blood pressure were repeatedly measured and recorded. In addition, clinical laboratory blood and urine tests were performed. Adverse events were recorded.

### Imaging and sampling

Subjects (mean age: 31 ± 10 years, mean weight: 81 ± 10 kg) underwent two consecutive 60‐minute dynamic [^11^C]elacridar (injected radioactivity: 394 ± 15 MBq, corresponding to 5.9 ± 1.8 μg of unlabeled elacridar) or [^11^C]tariquidar (injected radioactivity: 386 ± 19 MBq, corresponding to 5.8 ± 3.1 μg of unlabeled tariquidar) PET scans on a GE Advance scanner (General Electric Medical Systems, Milwaukee, WI) and serial arterial blood sampling as described previously.^26^ Selected plasma samples were analyzed for radiolabeled metabolites of [^11^C]elacridar or [^11^C]tariquidar using a previously described solid‐phase extraction protocol.^26^ An arterial plasma input function was constructed using total radioactivity counts in plasma as reported before.^26^ One hour before the start of the second PET scan an i.v. infusion containing 0.6 mg tariquidar free base per mL (AzaTrius Pharmaceuticals, Mumbai, India) was started at an infusion rate of 375 mL/h and maintained until the end of the scan as described before (total infusion length: 121 ± 4 minutes, total administered tariquidar dose: 5.7 ± 0.8 mg/kg).^18^ Three arterial blood samples (5 mL) were collected at the beginning, in the middle, and at the end of the second PET scan to measure plasma concentrations of tariquidar by a previously described liquid chromatography tandem mass‐spectrometry assay.^17^ Plasma protein binding of both radiotracers was determined with ultrafiltration as described before^42^ by incubating plasma samples collected immediately before each PET scan with either [^11^C]elacridar or [^11^C]tariquidar.

### PET data analysis and modeling

A whole brain gray matter region of interest was defined on individual magnetic resonance images acquired in all study participants (T1‐weighted Achieva 3.0T scanner; Philips Medical Systems, Best, The Netherlands) using the Hammersmith n30r83 3D maximum probability atlas of the human brain.^43^ In addition, the pituitary gland was manually delineated. TACs were extracted from the coregistered dynamic PET images as described previously.^44^ A standard 2T4K compartment model (**Supplementary Figure 3**) was fitted to the TACs from 0–60 minutes after radiotracer injection as described previously.^26^, ^44^ In addition, a 1T2K model (**Supplementary Figure 3**) was fitted to the first 10 minutes of the PET data as described before.^17^ In order to obtain a model‐independent estimate of *V*_T_, Logan graphical analysis was performed.^45^ Moreover, AUCs for brain and plasma TACs were calculated and the ratio between the brain AUC and plasma AUC, designated as AUCR, was calculated as an additional parameter of radiotracer brain distribution.

### Cell lines

LLC‐PK1 cells transduced with human ABCB1 (LLC‐ABCB1) and MDCK‐II cells transduced with human ABCG2 (MDCK‐ABCG2) were kindly provided by Prof. P. Borst and Dr. A. Schinkel (Netherlands Cancer Institute, Amsterdam, Netherlands). Culturing of the cells and testing of vincristine sulfate resistance (0.64 μmol/L) for LLC‐ABCB1 cells and mitoxantrone resistance (20 μmol/L) for MDCK‐ABCG2 cells was performed as described previously.^46^

### *In vitro* uptake assays

Cells were seeded with a density of 0.3 × 10^6^ cells/cm^2^ on 96‐well plates (white Isoplates for liquid scintillation counting, Perkin Elmer, Rodgau, Germany). Then 5–7 days after the cells reached 100% confluence, the experiment was initiated with 1‐hour preincubation at 37°C, 5% carbon dioxide, and 95% humidity, for which culture medium was replaced by serum‐free Opti‐MEM (Gibco/Life Technologies, Darmstadt, Germany). Solvent (dimethylsulfoxide) or tariquidar in concentrations of 5, 10, 20, 35, 50, 200, 5,000 nmol/L was added to the medium for LLC‐ABCB1 cells and in concentrations of 100, 200, 500, 1,000, 2,000, 5,000 nmol/L for MDCK‐ABCG2 cells. At the beginning of the experiment, medium was changed to Opti‐MEM containing either [^3^H]elacridar (provided as a gift by Albert D. Windhorst, VU University Medical Center, Amsterdam, The Netherlands) or [^3^H]tariquidar (purchased from American Radiolabeled Chemicals, St. Louis, MO) (both at 1 nmol/L) with unlabeled tariquidar or solvent at the same concentration as used for preincubation. After 120 minutes (37°C, 5% carbon dioxide, 95% humidity), cells were transferred on ice, washed twice with ice‐cold phosphate‐buffered saline before lysis buffer (25 mmol/L Tris, pH 8, 50 mmol/L sodium chloride, 0.5% (w/v) deoxycholic acid, 0.5% (w/v) Triton X‐100) supplemented with complete protease inhibitor (Roche, Mannheim, Germany) was added and protein concentrations in the respective wells were determined by using the Pierce BCA Protein Assay kit (ThermoScientific, Pittsburgh, PA). The amount of [^3^H]elacridar or [^3^H]tariquidar in the lysates was quantified with a liquid β‐scintillation‐counter (Microbeta Trilux, Perkin Elmer).

### Analysis of *in vitro* uptake data

Normalized response to ABCB1 or ABCG2 inhibition was obtained by subtracting the mean uptake (pmol elacridar or tariquidar/mg protein) without tariquidar from the individual uptake values with different tariquidar concentrations followed by normalization to the mean uptake with maximum ABCB1 (200 nmol/L tariquidar) or maximum ABCG2 inhibition (5,000 nmol/L tariquidar). With Prism 6.0 software (GraphPad Software, La Jolla, CA), a sigmoidal model with variable slope was used to analyze concentration–response relationships by nonlinear regression.

### Statistical analysis

All data are given as mean ± standard deviation (SD). Differences in outcome parameters between scan 1 and 2 were tested using the Wilcoxon matched‐pairs signed rank test and differences between groups were tested with the Mann–Whitney *U*‐test (Statistica 6.1, StatSoft, Tulsa, OK). Correlations were assessed by calculating Spearman's correlation coefficients using Prism 6.0 software. *P* < 0.05 was considered statistically significant.

## CONFLICT OF INTEREST

The authors declare that they have no conflicts of interest.

## AUTHOR CONTRIBUTIONS

O.L., M.B., and K.R. wrote the article; O.L., M.B., K.R., W.L., M.H., and M.Z. designed the research; M.B., K.R., B.W., J.S., C.P., A.M.‐S., H.H, C.J., W.W., W.J., and O.L. performed the research; M.B., K.R., R.K., W.L., M.Z., and O.L. analyzed the data.

## Supporting information

Supporting Information

Supporting Information

Supporting Information

Supporting Information

Supporting Information

Supporting Information

Supporting Information
