# Supplementary material for: Pilot PET Study to Assess the Functional Interplay Between ABCB1 and ABCG2 at the Human Blood–Brain Barrier
Source: Clin Pharmacol Ther. 2016 May 9;100(2):131–41. doi: 10.1002/cpt.362 (PMC4979595; doi:10.1002/cpt.362)
Supplement: Supplementary file 1 — Supporting Information [file CPT-100-131-s001.docx]

**Supplementary Table 1 Areas under the curve (AUC) for plasma time-activity curves of [^11^C]elacridar and [^11^C]tariquidar in c.421CC and c.421CA subjects for baseline scan and scan during ABCB1 inhibition with tariquidar**

| Group | AUC  baseline | AUC ABCB1 inhibition | % change over baseline | *p* |
| --- | --- | --- | --- | --- |
| [^11^C]elacridar c.421CC | 37.2 ± 6.2 | 46.9 ± 8.0 | 27 ± 12 | 0.043 |
| [^11^C]tariquidar c.421CC | 36.5 ± 6.7 | 43.5 ± 6.5 | 21 ± 18 | 0.043 |
| [^11^C]tariquidar c.421CA | 41.5 ± 15.8 | 44.9 ± 8.6 | 16 ± 30 | 0.686 |

AUC values are given as mean ± standard deviation averaged over all subjects (*n* = 5) per group. AUC values were calculated from 0 to 60 min after radiotracer injection and are given in units of SUV x min (SUV, standardized uptake value). *P* values for comparison with baseline scan using Wilcoxon matched-pairs signed rank test are also stated.
