# Supplementary material for: Pilot PET Study to Assess the Functional Interplay Between ABCB1 and ABCG2 at the Human Blood–Brain Barrier
Source: Clin Pharmacol Ther. 2016 May 9;100(2):131–41. doi: 10.1002/cpt.362 (PMC4979595; doi:10.1002/cpt.362)
Supplement: Supplementary file 2 — Supporting Information [file CPT-100-131-s002.docx]

**Supplementary Table 2 Tariquidar concentrations in plasma of individual subjects (µmol/L) during ABCB1 inhibition PET scan**

| **Time** | **Subject 1** | **Subject 2** | **Subject 3** | **Subject 4** | **Subject 5** |
| --- | --- | --- | --- | --- | --- |
|  | **[^11^C]elacridar** **c.421CC** | | | | |
| **start of PET** | 2.67 | 1.83 | 1.71 | 2.74 | 2.73 |
| **middle of PET** | 2.33 | 2.55 | 2.71 | 2.43 | 3.73 |
| **end of PET** | 2.45 | 1.98 | 2.58 | 2.72 | 4.03 |
|  | **[^11^C]elacridar** **c.421CA** | | | | |
| **start of PET** | 1.94 *^a^* |  |  |  |  |
| **middle of PET** | 2.23 |  |  |  |  |
| **end of PET** | 2.22 |  |  |  |  |
|  | **[^11^C]tariquidar c.421CC** | | | | |
| **start of PET** | 2.91 | 2.17 | 2.65 | 2.67 | 2.64 |
| **middle of PET** | 2.68 | 2.09 | 2.95 | 2.70 | 2.65 |
| **end of PET** | 2.71 | 2.78 | 3.13 | n.d. | 3.70 |
|  | **[^11^C]tariquidar c.421CA** | | | | |
| **start of PET** | 3.70 | 2.61 | 2.87 *^a^* | 2.47 | 2.80 |
| **middle of PET** | 3.93 | 3.55 | 3.31 | 2.86 | 3.57 |
| **end of PET** | 3.67 | 3.18 | 2.24 | 3.08 | 3.79 |
|  | **(*R*)-[^11^C]verapamil** *^b^* | | | | |
| **start of PET** | 2.81 | 1.70 | 2.36 | 1.91 | 2.11 |
| **middle of PET** | 3.61 | 2.71 | 3.88 | 2.85 | 2.56 |
| **end of PET** | 3.76 | 2.52 | 4.11 | 2.83 | 3.38 |

*^a^* Identical subject

*^b^* data from previous study,^[18](#_ENREF_18)^ no *ABCG2* genotype of subjects available

n.d., not determined
