# Supplementary material for: Pilot PET Study to Assess the Functional Interplay Between ABCB1 and ABCG2 at the Human Blood–Brain Barrier
Source: Clin Pharmacol Ther. 2016 May 9;100(2):131–41. doi: 10.1002/cpt.362 (PMC4979595; doi:10.1002/cpt.362)
Supplement: Supplementary file 3 — Supporting Information [file CPT-100-131-s003.docx]

**Supplementary Table 3 Fraction transported (f_t_)*^a^* determined for [^11^C]elacridar, [^11^C]tariquidar and (*R*)-[^11^C]verapamil based on *V*_T_ (Logan) values in whole brain gray matter and pituitary gland**

| **Radiotracer/group** | **f_t_ baseline** | **f_t_ ABCB1 inhibition** |
| --- | --- | --- |
| [^11^C]elacridar/c.421CC | 0.92 ± 0.03 | 0.87 ± 0.03 *^b^* |
| [^11^C]tariquidar/c.421CC | 0.93 ± 0.02 | 0.92 ± 0.03 |
| [^11^C]tariquidar/c.421CA | 0.89 ± 0.02 | 0.78 ± 0.09 |
| (*R*)-[^11^C]verapamil | 0.79 ± 0.07 | 0.15 ± 0.12 *^b^* |

*^a^* F_t_ is calculated as 1-1/(*V*_T,pituitary_/*V*_T,brain_)

*^b^* *P* < 0.05 for comparison with baseline scan using Wilcoxon matched-pairs signed rank test
