# Supplementary figures and images for: Pilot PET Study to Assess the Functional Interplay Between ABCB1 and ABCG2 at the Human Blood–Brain Barrier
Source: Clin Pharmacol Ther. 2016 May 9;100(2):131–41. doi: 10.1002/cpt.362 (PMC4979595; doi:10.1002/cpt.362)

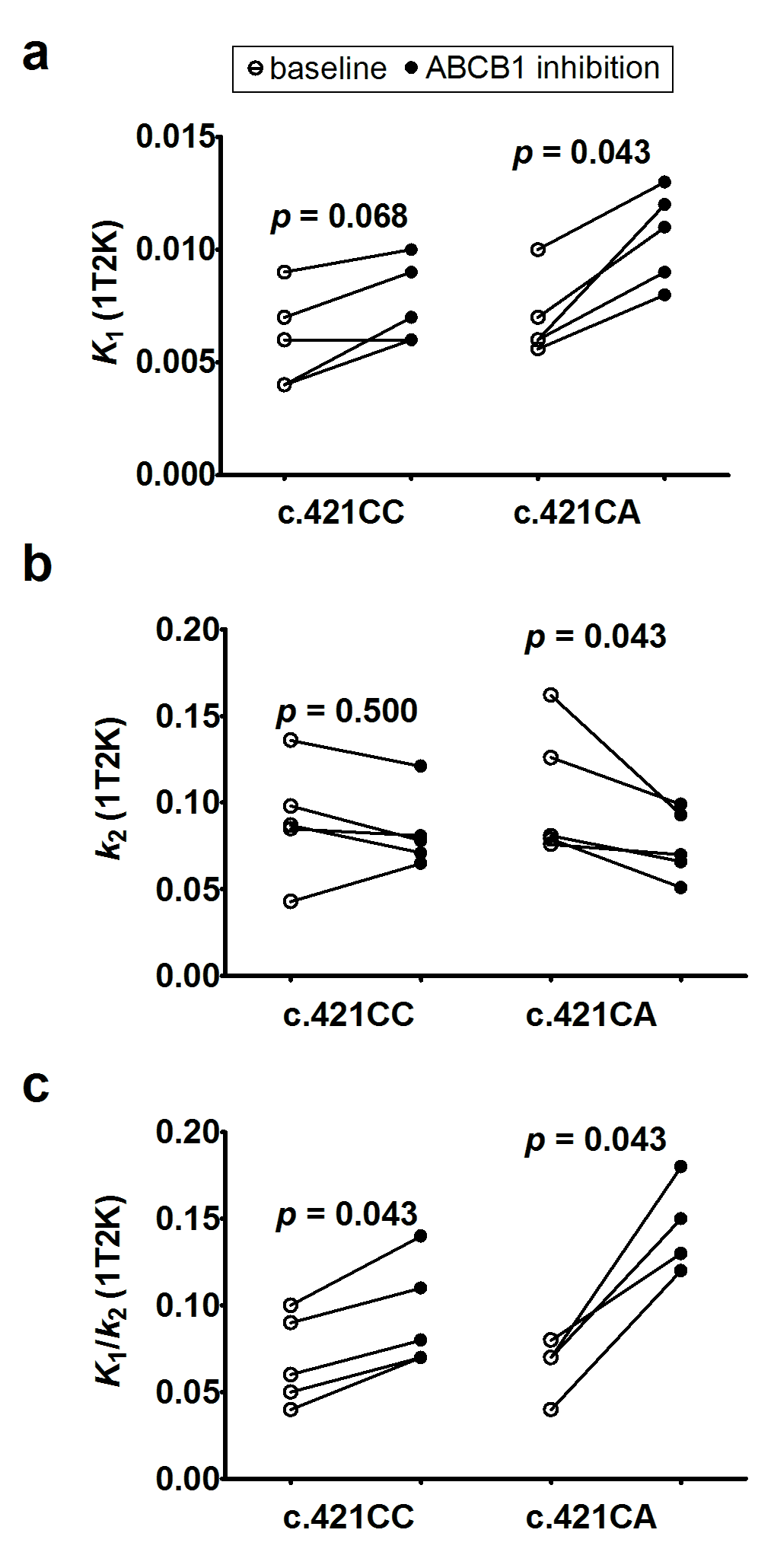

Supplement: Supplementary file 4 — Supporting Information [file CPT-100-131-s004.tif]

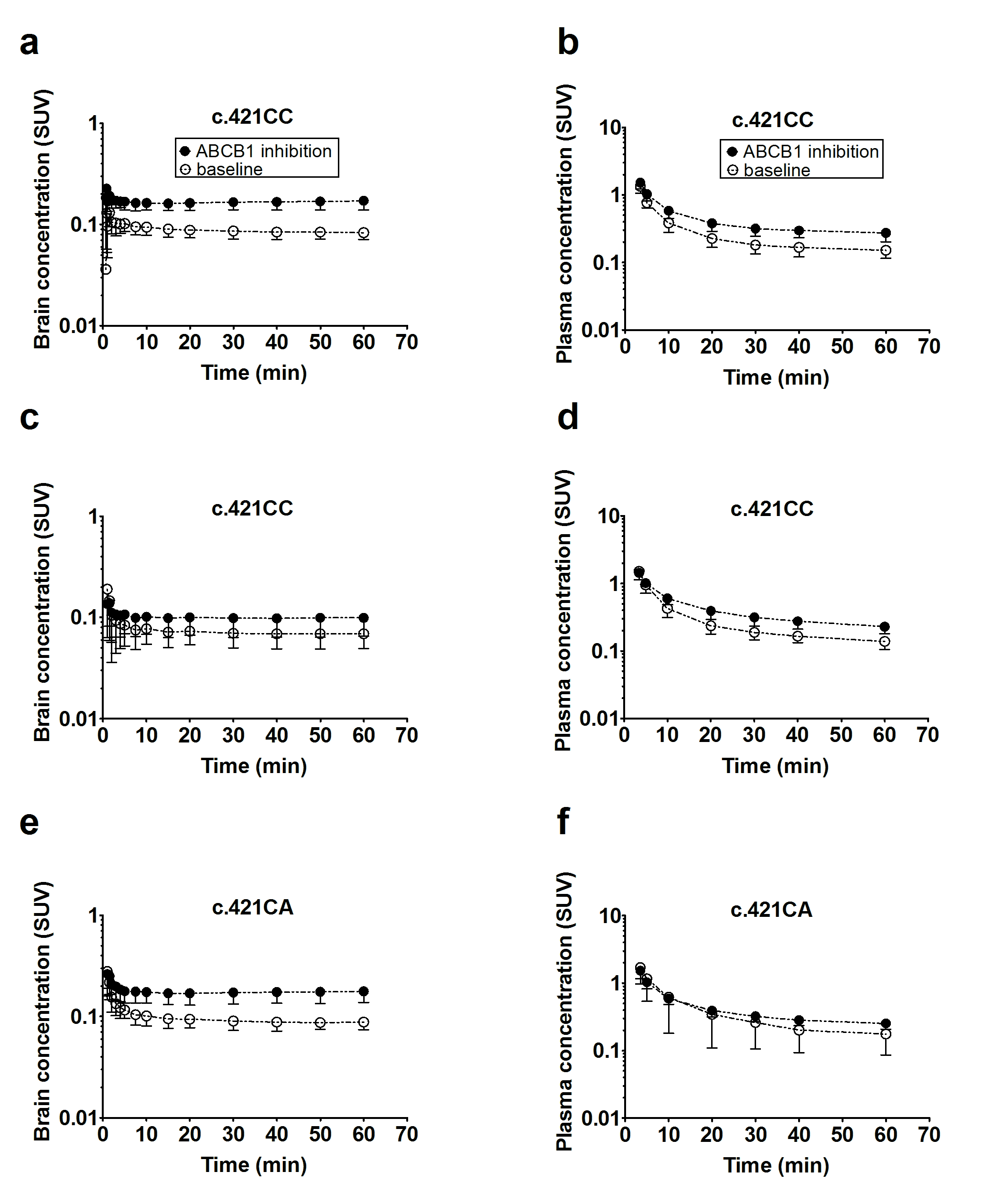

Supplement: Supplementary file 5 — Supporting Information [file CPT-100-131-s005.tif]

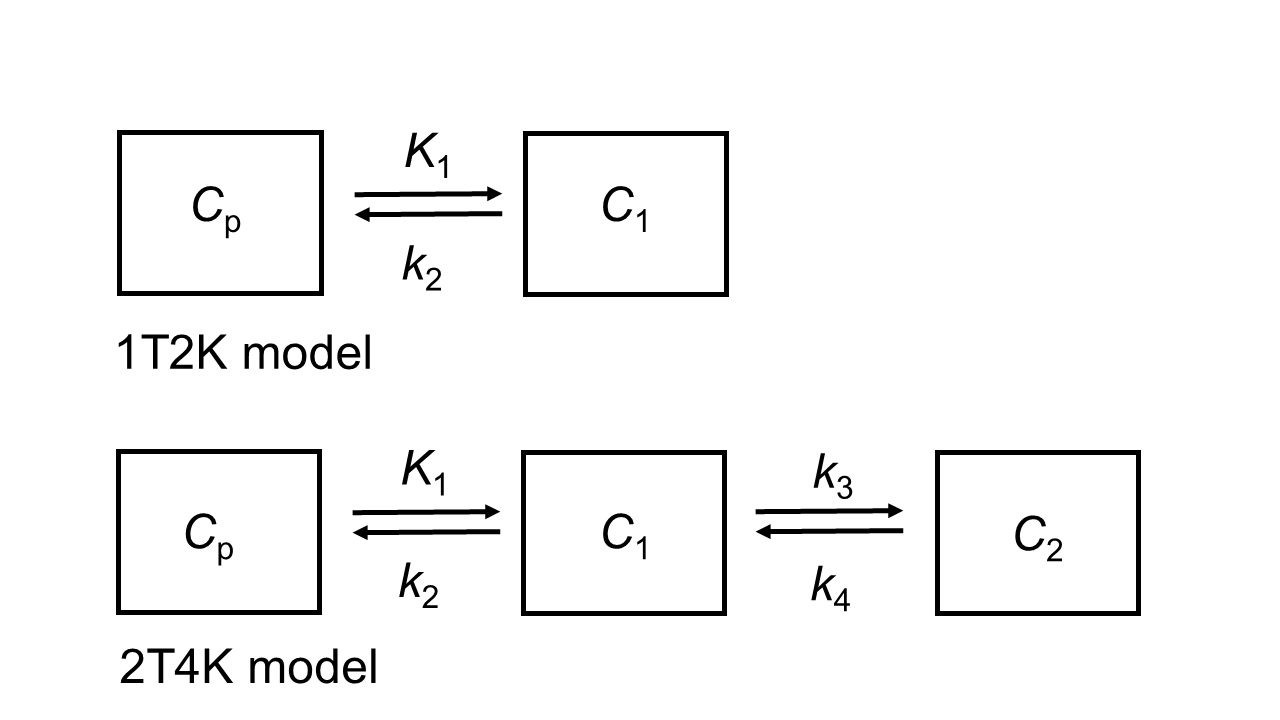

Supplement: Supplementary file 6 — Supporting Information [file CPT-100-131-s006.tif]
